# Current *Helicobacter pylori* infection is significantly associated with subclinical coronary atherosclerosis in healthy subjects: A cross-sectional study

**DOI:** 10.1371/journal.pone.0193646

**Published:** 2018-03-02

**Authors:** Minyoung Lee, Haeri Baek, Jong Suk Park, Sohee Kim, Chanhee Kyung, Su Jung Baik, Byoung Kwon Lee, Jie-Hyun Kim, Chul Woo Ahn, Kyung Rae Kim, Shinae Kang

**Affiliations:** 1 Division of Endocrinology, Department of Internal Medicine, Gangnam Severance Hospital, Yonsei University College of Medicine, Seoul, Korea; 2 Department of Internal Medicine, H-plus Yangji General Hospital, Seoul, Korea; 3 Severance Institute for Vascular and Metabolic Research, Yonsei University College of Medicine, Seoul, Korea; 4 Aswell convalescent hospital, Gwangju, Korea; 5 Rhin Hospital, Gyeonggi-do, Korea; 6 Healthcare Research Team, Health Promotion Center, Gangnam Severance Hospital, Seoul, Korea; 7 Division of Cardiology, Department of Internal Medicine Gangnam Severance Hospital, Yonsei University College of Medicine, Seoul, Korea; 8 Division of Gastroenterology, Department of Internal Medicine, Gangnam Severance Hospital, Yonsei University College of Medicine, Seoul, Korea; University Hospital Llandough, UNITED KINGDOM

## Abstract

*Helicobacter pylori* is a gastrointestinal pathogen known to be associated with cardiovascular disease (CVD). However, most analyses about the effect of *H*. *pylori* infection have been done in patients with a history of CVD but not in healthy subjects. We evaluated the association between *H*. *pylori* infection and subclinical atherosclerosis by using cardiac multidetector computed tomography (MDCT) in healthy subjects without previous CVD. From December 2007 to February 2014, 463 subjects who underwent the rapid urease test (CLO test), pulse-wave velocity (PWV) measurement, and MDCT for a self-referred health check-up were enrolled to this study. *Helicobacter pylori* infection was defined on the basis of CLO test positivity on endoscopic gastric biopsy. Significant coronary artery stenosis was defined as ≥50% stenosis in any of the major epicardial coronary vessel on MDCT. The CLO-positive subjects had a lower high-density lipoprotein-cholesterol (HDL-cholesterol) level compared to the CLO-negative subjects. The incidence of significant coronary stenosis was higher in the CLO-positive group (7.6% vs. 2.9%, *P* = 0.01). Furthermore, the number of subjects with coronary artery calcium score >0 and log{(number of segments with plaque)+1} were also significantly higher in the CLO-positive group. However, there was no statistical difference in the number of subjects with coronary artery calcium score >100, the prevalence of any plaque nor the plaque characteristics (calcified, mixed, or soft). Pulse-wave velocity (PWV) was neither associated with CLO test positivity. The CLO-positive group was 3-fold more likely to have significant coronary artery stenosis even after adjusting for confounding factors (adjusted odds ratio 2.813, 95% confidence interval 1.051–7.528, *P* = 0.04). In a healthy population, current *H*. *pylori* infection was associated with subclinical but significant coronary artery stenosis. The causal relationship between *H*. *pylori* infection and subclinical atherosclerosis in a “healthy” population remains to be investigated in the future.

## Introduction

Several studies have reported that cardiovascular disease (CVD) may be associated with certain microorganisms such as *Chlamydia pneumonia*, cytomegalovirus, or *Helicobacter pylori* [1]. Those reports have suggested that the pathogens might directly invade the vessel wall, leading to localized vascular inflammation [2]. Additionally, not only localized but also systemic inflammation by such pathogens might indirectly induce endothelial dysfunction and dyslipidemia, resulting in CVD [3].

*Helicobacter pylori* is a gram-negative bacterium that lives in the stomach and a carcinogen leading to stomach cancer [4,5]. The infection rate of *H*. *pylori* is especially high in Asians [6]. Because local and systemic inflammation by microbes and infectious agents is known to be crucial in CVD [7,8], the relationship between *H*. *pylori* infection and CVD has received considerable attention especially in Asian countries [9–12].

*H*. *pylori* has been suggested as a possible contributor to CVD progression but the results from the previous studies were quite controversial. Some reports showed that the seroprevalence of *H*. *pylori* was not correlated with coronary artery disease and they insisted that the infection status of *H*. *pylori* did not determine the risk of CVD [13–16]. In contrast, other reports demonstrated a meaningful higher prevalence of *H*. *pylori* infection among patients with previous history of CVD and supported a possible connection between *H*. *pylori* and the development of CVD [12,17–21]. In healthy populations without previous CVD, however, it still remains unclear whether *H*. *pylori* infection is significantly associated with subclinical atherosclerosis. The individuals with subclinical atherosclerosis are at a higher risk of developing clinical atherosclerosis including coronary heart disease compared to the individuals without subclinical diseases [22–24]. If the association between *H*. *pylori* and subclinical atherosclerosis is significant, earlier diagnosis and possibly eradication of *H*. *pylori* might be necessary for preventing atherosclerosis progression, especially in the high-risk population. Additionally, most studies on *H*. *pylori* infection and CVD have relied on antibody tests for defining the *H*. *pylori* infection status [17,18,20], the results of which cannot differentiate between current versus past infections. Additionally, tests using the serum antibodies demonstrate lower specificity than that of the rapid urease test (CLO test) [25,26].

In this study, we aimed to evaluate whether current *H*. *pylori* infection, diagnosed by using an endoscopic CLO test, was associated with subclinical atherosclerosis using pulse-wave velocity (PWV) and cardiac multidetector computed tomography (MDCT) in a healthy population.

## Methods

### Study design and population

**Fig 1:**
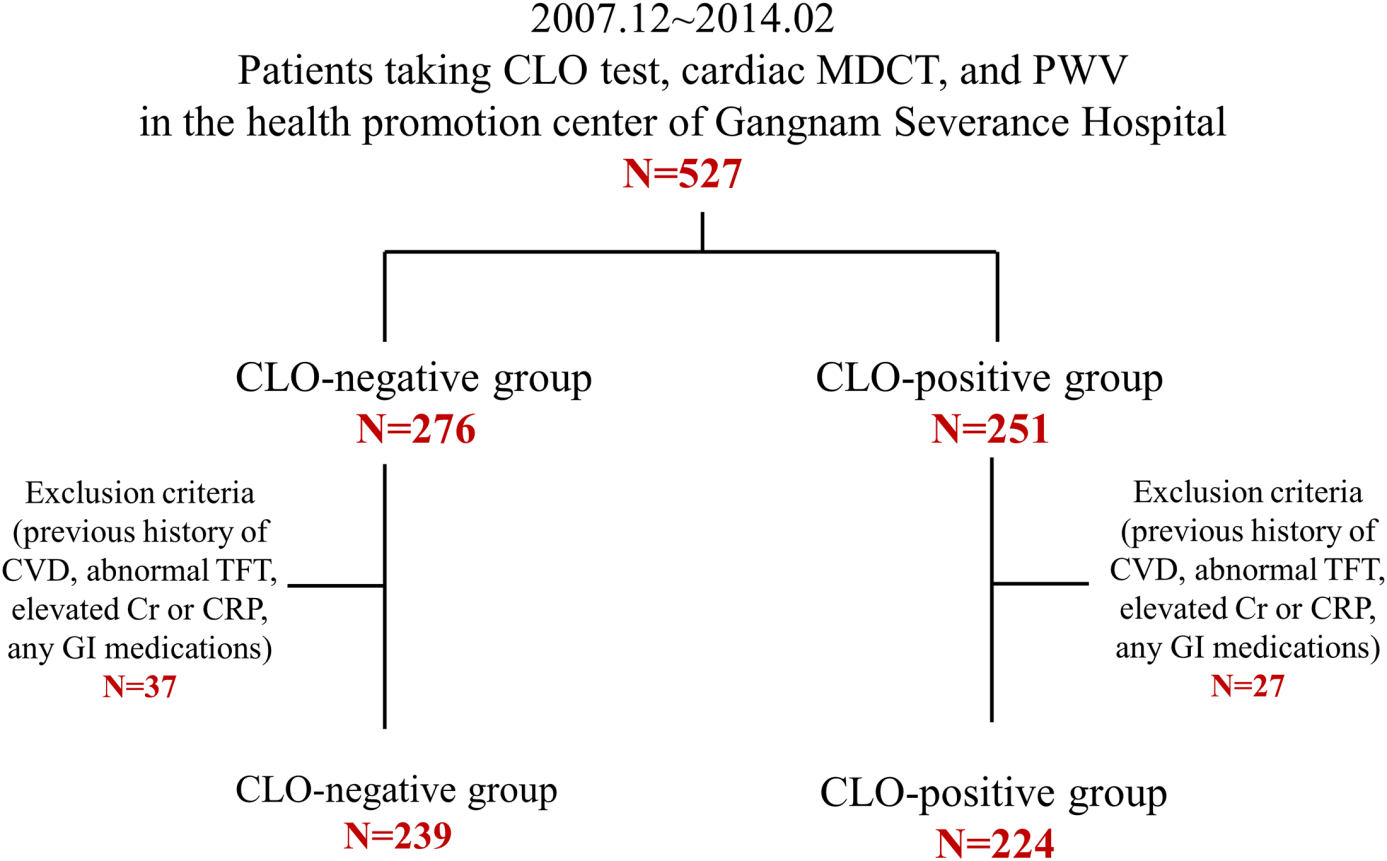
Schematic diagram and overall flow of study participants. Abbreviations: CLO test, rapid urease test; cardiac MDCT, cardiac multidetector computed tomography; PWV, pulse-wave velocity; CVD, cardiovascular disease; TFT, thyroid function test; Cr, creatinine; CRP, C-reactive protein; GI medications, gastrointestinal medications.

This study included 527 consecutive subjects undergoing esophagogastroduodenoscopy with the CLO test, PWV, and MDCT as part of the self-referred health check-up program at Gangnam Severance Hospital Health Promotion Center, Seoul, Korea from December 2007 to February 2014. All participants were divided into two study groups according to the CLO positivity. To avoid confounding bias, we excluded possible confounding medical conditions known to be associated with the health outcome. The exclusion criteria were as follows: (i) history of coronary artery disease, (ii) significant hypothyroidism/hyperthyroidism (thyroid-stimulating hormone >4.5 μIU/mL or free thyroxine >2.0 ng/dL), (iii) significant renal insufficiency (creatinine >1.5 mg/dL), (iv) significant CRP (C-reactive protein) elevation (>10.0 mg/L) and (v) subjects with any GI (gastrointestinal) medications. Since we intended to investigate the association between *H*. *pylori* infection and subclinical atherosclerosis in healthy population, subjects with previous coronary artery disease were excluded. Subjects with thyroid dysfunction and renal insufficiency were omitted as changes in thyroid or renal function influence the level of serum lipids along with coronary atherosclerosis [27–29]. We excluded the subjects with elevated CRP levels as various pro-inflammatory conditions and diseases can affect the progression of atherosclerosis [30,31]. We further excluded the subjects taking GI medications for gastritis or gastric ulcer. Finally, 463 participants were included in the current analysis (Fig 1). The protocol of this study was approved by the institutional review board of Gangnam Severance Hospital.

### Anthropometric and biochemical measurements

We reviewed the participants’ questionnaire at the time of the health check-up, which included medical history, concomitant medication use, and other medico-social history information. After an overnight fasting for ≥8 hours, the fasting plasma glucose, total cholesterol, HDL-cholesterol, triglyceride, calcium, and C-reactive protein levels were checked from the peripheral venous blood samples by means of adequate enzymatic methods (Hitachi 7600–120 automated chemistry analyzer; Hitachi, Tokyo, Japan). The Friedewald formula was used to calculate the low-density lipoprotein-cholesterol (LDL-cholesterol) level.

### Coronary artery calcium score, coronary artery stenosis, and coronary plaque measurement with MDCT

Coronary artery calcium scores (CACSs), coronary artery stenosis, and the number, presence and the characteristics of intracoronary plaques were evaluated by using an MDCT scanner (Philips Brilliance 64; Philips Medical System, Best, the Netherlands). A standard prospective electrocardiogram-gating protocol with a step-and-shoot technique (64 × 0.625 mm slice section collimation, 420-ms rotation time, 120-kV tube voltage, and 210-mAs tube current) was used. The CACS was measured (Extended Brilliance Workspace BW V4.5.2.4031, Philips Medical System) and described as Agatston scores. The CACS was interpreted as having either no coronary calcium (CACS 0 vs. >0) or severe coronary calcium (CACS ≤100 vs. >100). Coronary arteries were segmented to 3 major arteries and 12 small branches. Significant coronary artery stenosis was defined as at least ≥50% stenosis in any coronary artery/branch. A plaque was defined as a structure >1 mm^2^ within and/or adjacent to the vessel lumen, and classified according to the presence/proportion of intraplaque calcification. The plaques were divided into calcified (calcium [>130 Hounsfield units] content ≥50% of the whole plaque), mixed (calcium content <50% of the whole plaque) or soft (no calcium content). Log{(number of segments with plaque) + 1} was used to analyze the number of segments with plaque.

### Pulse-wave velocity

Brachial-ankle PWV (ba-PWV) was assessed by using a volume plethysmographic instrument (VP-1000; Omron Healthcare Corporation, Kyoto, Japan). The ba-PWV was estimated as the value of brachial-ankle distance divided by the blood transit time. An automatic device recorded electrocardiograms, phonocardiograms and the blood pressure (BP) at both the brachial artery and the posterior tibial artery after a stable rest for ≥5 min in supine position. The average value of the right and left sides was defined as the mean PWV.

### Rapid urease test

Biopsy of the gastric mucosa was performed to assess the *H*. *pylori* infection status with a commercial CLO kit (ASAN Pharm. Co., Seoul, Korea). Each gastric mucosal biopsy specimen was immediately transferred into the CLO kit and analyzed after 30 min of reaction to determine the test positivity according to the color change of the reaction kit.

### Statistical analyses

Continuous variables with normal distribution are presented as mean ± standard error (SE), and those with a skewed distribution, such as CACS or the number of coronary segments with any plaque, were log transformed for analyses. Categorical variables are presented as absolute numbers and percentages. Intergroup comparisons with age adjustment were performed by using analysis of covariance for continuous variables and the generalized estimating equation for categorical variables. Adjusted odds ratios (ORs) for significant coronary artery stenosis in the CLO-positive group compared with the negative group were estimated with logistic regression analysis models. The SPSS statistical package (version 20.0; SPSS Inc., Chicago, IL, USA) was used for all statistical analyses. A *P*-value of ≤0.05 was considered statistically significant.

## Results

### Clinical and biochemical characteristics of the study population

A total of 463 subjects (336 men, 127 women, mean age 54.2 ± 8.5 years) were analyzed for the current study and divided into two groups according to the CLO test result. There were 224 (48.4%) CLO-positive and 239 (51.6%) CLO-negative subjects, respectively. The clinical and biochemical characteristics of the study subjects are presented in Table 1. The minimum and maximum age of this whole study population was 31 and 80 years, respectively (33–80 years for the CLO-negative group; and 31–77 years for the CLO-positive group). The mean age was different between the two groups (53.2 ± 7.9 years for the CLO-positive group vs. 55.3 ± 8.9 years for the CLO-negative group, *P* = 0.01). Therefore, comparisons throughout this study were performed after adjustment for age as a confounding variable. The CLO-positive group had a lower mean HDL-cholesterol level (46.6 ± 0.8 mg/dL vs. 49.6 ± 0.8 mg/dL, *P* = 0.01) and a higher mean triglyceride level (134.5 ± 5.7 mg/dL vs. 116.4 ± 5.5 mg/dL, *P* = 0.02). There were no differences in the prevalence of diabetes and hypertension nor the use of antidiabetic, antihypertensive, and antiplatelet agents between the two groups. Subjects in the CLO-positive group were more likely to take a lipid-lowering agent compared to those in the CLO-negative group (15.6% vs. 10.5%, *P* = 0.05).

**Table 1:** Clinical and biochemical characteristics of study subjects with age adjustment.

	Total N = 463	CLO-negative N = 239	CLO-positive N = 224	*P*-value
**Demographics**				
Age (yr)	54.2 ± 8.5	55.3 ± 8.9	53.2 ± 7.9	0.01
minimum and maximum	31–80	33–80	31–77	
Male (N, %)	336 (72.6)	165 (69.0)	171 (76.3)	0.07
Hypertension (N, %)	213 (46.0)	113 (47.3)	100 (44.6)	0.89
Diabetes (N, %)	79 (17.1)	39 (16.3)	40 (17.9)	0.35
Diabetes medication (N, %)	46 (9.9)	27 (11.3)	19 (8.5)	0.58
Lipid lowering agent (N, %)	60 (13.0)	25 (10.5)	35 (15.6)	0.05
Antiplatelet agent (N, %)	76 (16.4)	42 (17.6)	34 (15.2)	0.84
**Anthropometrics**				
Systolic BP (mmHg)	128.2 ± 16.7	128.2 ± 1.1	128.2 ± 1.1	0.97
Diastolic BP (mmHg)	80.1 ± 9.9	79.8 ± 0.6	80.4 ± 0.7	0.51
BMI (Kg/m^2^)	24.3 ± 3.1	24.1 ± 0.2	24.5 ± 0.2	0.26
**Laboratory indices**				
Fasting glucose (mg/dL)	100.6 ± 24.8	98.9 ± 1.6	102.5 ± 1.7	0.12
Total cholesterol(mg/dL)	191.3 ± 36.3	193.2 ± 2.3	189.3 ±2.4	0.25
Triglyceride (mg/dL)	125.2 ± 84.8	116.4 ± 5.5	134.5 ± 5.7	0.02
HDL-cholesterol (mg/dL)	48.2 ± 12.5	49.6 ± 0.8	46.6 ± 0.8	0.01
LDL-cholesterol (mg/dL)	116.8 ± 32.9	119.1 ± 2.1	114.3 ± 2.2	0.12
Calcium (mg/dL)	9.1 ± 0.52	9.1 ± 0.03	9.0 ± 0.03	0.37
CRP (mg/L)	1.2 ± 1.32	1.2 ± 0.09	1.2 ± 0.09	0.77

BP, blood pressure; BMI, body mass index; HDL-cholesterol, high density lipoprotein-cholesterol; LDL-cholesterol, low density lipoprotein-cholesterol; CRP, C-reactive protein; DM, diabetes mellitus. A one-way analysis of variance was used to evaluate the difference of continuous variables between CLO positive and negative subjects with age adjustment. The generalized estimating equation was used to compare categorical variables between the CLO positive and negative groups with age adjustment. Continuous variables are presented as mean±standard error or the value of minimum and maximum. Dichotomous variables are presented as the number of subjects with the percentage of subjects in the parenthesis. P < .05 was regarded as statistically significant.

### Differences in PWV, CACS, and incidence of coronary stenosis/intracoronary plaque between the CLO-positive versus the CLO-negative subjects

The presence of coronary artery calcium (CACS >0) was significantly higher in the CLO-positive group (36.7% vs. 32.5%, *P* = 0.05) than in the CLO-negative group. However, there were no statistical differences in the presence of severe coronary artery calcium (CACS >100), the mean value of log(CACS + 1) or the mean PWV between the CLO-positive and CLO-negative subjects. There was also no difference between the two groups not only in the prevalence of any plaque but also in the characteristics of plaque (calcified, mixed, or soft). However, the number of segments with plaque was higher in the CLO-positive group (0.22 ± 0.02 vs. 0.17 ± 0.02, *P* = 0.03). More importantly, the incidence of significant coronary artery stenosis (≥50% stenosis of any major vessel) was significantly higher in the CLO-positive subjects than in the CLO-negative subjects (7.6% vs. 2.9%, *P* = 0.01) (Table 2).

**Table 2:** Difference of subclinical atherosclerosis between the CLO-negative and CLO-positive subjects with age adjustment.

	Total N = 463	CLO-negative N = 239	CLO-positive N = 224	*P*-value
**PWV (cm/s)**	1422.4 ± 234.3	1419.9 ± 13.1	1425.0 ± 13.5	0.79
**CACS**				
CACS >0 (n, %)	157 (34.5)	76 (32.5)	81 (36.7)	0.05
CACS >100 (n, %)	51 (11.2)	27 (11.5)	24 (10.9)	0.49
Log (CACS +1)	0.57 ± 0.87	0.51 ± 0.05	0.64 ± 0.05	0.08
**Plaque**				
Any plaque (%)	104 (22.5)	52 (21.8)	52 (23.2)	0.32
Log{(Number of segments with plaque)+1}	0.20 ± 0.26	0.17 ± 0.02	0.22 ± 0.02	0.03
Calcified plaque (%)	58 (12.5)	29 (12.1)	29 (12.9)	0.38
Mixed plaque (%)	41 (8.9)	19 (7.9)	22 (9.8)	0.25
Soft plaque (%)	32 (6.9)	17 (7.1)	15 (6.7)	0.99
**Coronary artery stenosis (n, %)**	24 (5.2)	7 (2.9)	17 (7.6)	0.01

PWV, pulse-wave velocity; CACS, coronary artery calcium score. A one-way analysis of variance was used to evaluate the difference of PWV or number of coronary segments involved between CLO negative and positive subjects with age adjustment. The generalized estimating equation was used to evaluate the difference of dichotomous variables between the two groups with age adjustment. Continuous variables are presented as mean±standard error. Dichotomous variables are presented as the number of subjects with the percentage of subjects in the parenthesis. P < .05 was regarded as statistically significant.

### Elevated adjusted risk for significant coronary artery stenosis in CLO-positive subjects

**Fig 2:**
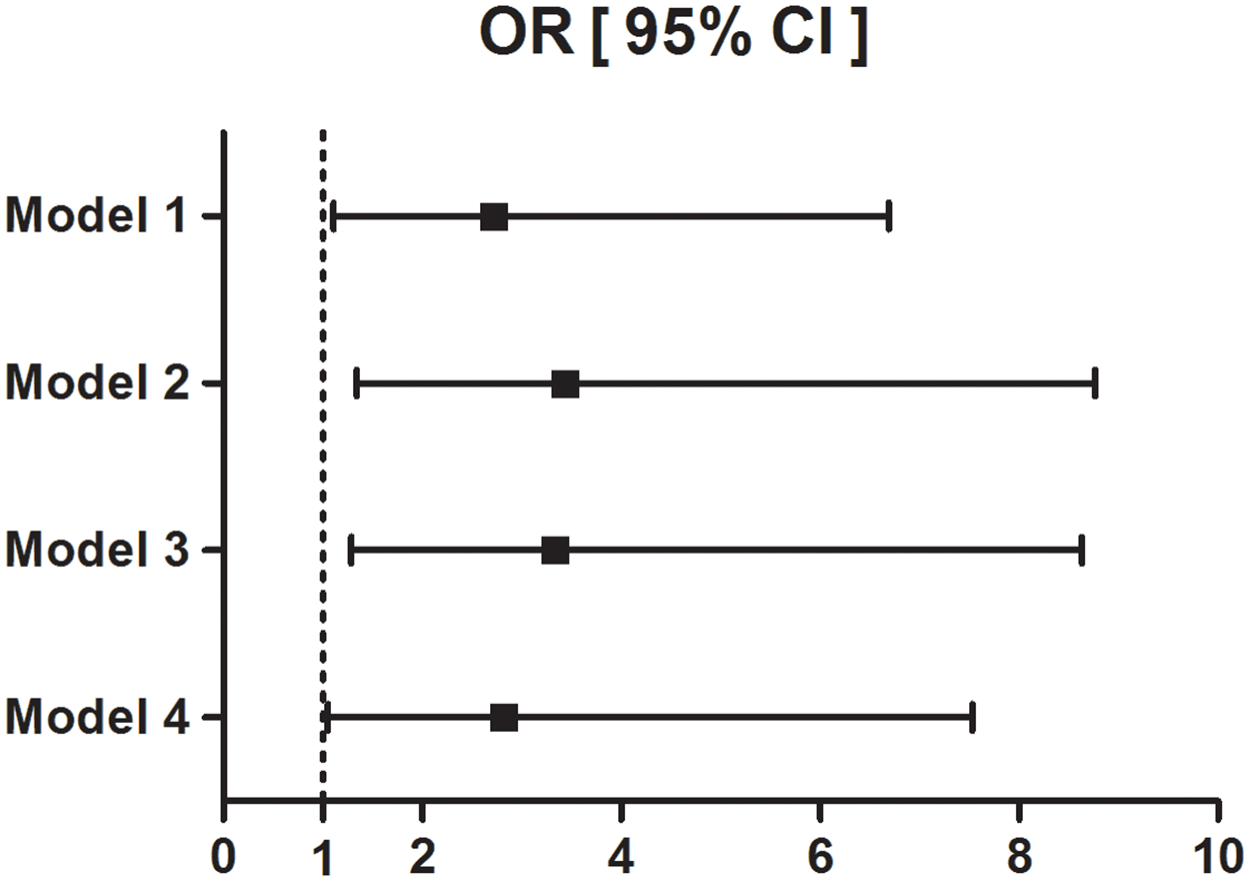
Odds ratio for significant coronary artery stenosis according to the CLO test. Logistic regression was used for calculating odds ratios with 95% confidence intervals. The reference group comprised the CLO-negative subjects. Model 1: Not adjustedModel 2: adjusted for age and sexModel 3: adjusted for age, sex, HDL-cholesterolModel 4: adjusted for age, sex, systolic BP, fasting glucose, HDL-cholesterol, anti-hypertension medication, anti-diabetic medication, lipid lowering agent, anti-platelet agent Abbreviations: OR, odds ratio; CI, confidence interval; BP, blood pressure; HDL-cholesterol, high density lipoprotein-cholesterol.

To investigate whether current *H*. *pylori* infection is an independent risk factor for subclinical atherosclerosis, we evaluated the adjusted risk for significant coronary artery stenosis in the CLO-positive subjects compared with the CLO-negative subjects by analyzing odds ratio (Table 3 and Fig 2). The CLO-positive subjects were 2.72-fold more likely to have significant coronary artery stenosis compared to the CLO-negative subjects. This risk became more significant after adjusting for age and sex (adjusted OR 3.431, 95% confidence interval 1.343–8.765, *P* = 0.01) and also after adjusting for several additional factors that may influence coronary artery stenosis, such as systolic blood pressure (BP), fasting glucose, HDL-cholesterol, anti-hypertension/diabetic medications, lipid-lowering agents, and antiplatelet agents (adjusted OR 2.813, 95% confidence interval 1.051–7.528, *P* = 0.04).

**Table 3:** Odds ratio for significant coronary artery stenosis.

	Odds ratio (95% CI)		*P*-value
	CLO-negative N = 239	CLO-positive N = 224	
Model 1	1.000 (reference)	2.722 (1.107–6.694)	0.03
Model 2		3.431 (1.343–8.765)	0.01
Model 3		3.330 (1.285–8.629)	0.01
Model 4		2.813 (1.051–7.528)	0.04

Model 1: Not adjusted Model 2: adjusted for age and sex Model 3: adjusted for age, sex, HDL-cholesterol Model 4: adjusted for age, sex, systolic BP, fasting glucose, HDL-cholesterol, anti-hypertension medication, anti-diabetic medication, lipid lowering agent, anti-platelet agent

CI, confidence interval; BP, blood pressure; HDL-cholesterol, high density lipoprotein-cholesterol. Data are presented as odds ratios with the CLO-negative group as a reference.

## Discussion

In this study, we demonstrated that current *H*. *pylori* infection is significantly associated with significant coronary artery stenosis in a healthy population. The elevated risk of coronary artery stenosis in *H*. *pylori*-infected subjects was associated with a lower HDL-cholesterol level. This is the first report demonstrating that active/current *H*. *pylori* infection can be a risk for subclinical atherosclerosis in healthy subjects without previous CVD.

The diagnostic tests for *H*. *pylori* can be divided into invasive and noninvasive methods. The serologic *H*. *pylori* IgG test, urea breath test, and stool antigen assay are noninvasive, whereas the CLO test can only be performed with stomach tissue obtained from invasive endoscopic biopsy [25]. As the serologic *H*. *pylori* IgG test is fast, cheap, and noninvasive, most of the studies on *H*. *pylori* infection and CVD were performed using this test [17,18,20,32–35]. However, conflicting results exist between the serology and the prevalence of CVD, with some studies showing a significant association between *H*. *pylori* seropositivity and CVD [12,17,18,20,32] while others showing no such association [33–35]. One of the possible reasons for these conflicting results may be certain limitations of the serologic test in detecting *H*. *pylori* infection itself [36]. Most importantly, serologic antibody testing cannot distinguish current and past infection.[26] Owing to this limitation, the serologic test has been mostly used to investigate merely the association between the prevalence of *H*. *pylori* infection and CVD [18,32,37]. In contrast, a positive CLO test means a current infection [26]. If current *H*. *pylori* infection is associated with subclinical atherosclerosis, it can be inferred that the eradication of *H*. *pylori* infection might help prevent the progression of atherosclerosis. In this context, this study provides a theoretical basis for studying the effect of *H*. *pylori* eradication on preventing the future progression of atherosclerosis.

However, the CLO test is not without limitations. For example, although atrophic gastritis is explicitly associated with *H*. *pylori* in many cases, the CLO test may fail to detect the presence of *H*. *pylori* because of a decreased bacterial burden in atrophic gastritis, particularly in the presence of intestinal metaplasia [26]. Nevertheless, the CLO test can provide more accurate and reliable results than the serologic test [25]. The specificity of the CLO test is 95–100%, whereas the serologic test has variable specificity ranging from 76% to 96% [25]. Moreover, the CLO test has a higher sensitivity (80–95%) than the serologic test (75–85%) [26]. Hence, our data provide more accurate evidence for examining the association between *H*. *pylori* infection and CVD compared with previous studies with the serologic test.

Several studies have reported the correlation between the prevalence of *H*. *pylori* infection and overt coronary artery disease [11,17–21]. However, no study has demonstrated the association between current *H*. *pylori* infection and subclinical coronary atherosclerosis. This study is distinct from previous studies because we did not observe CVD events but the incidence of subclinical coronary atherosclerosis in a relatively large number of healthy subjects without previous CVD. Atherosclerosis is a pathologic process narrowing the coronary, cerebral, and peripheral arteries due to the formation of atheromatous plaques [38]. Studying the association between *H*. *pylori* infection and subclinical atherosclerosis allows investigators to confirm that earlier vessel-wall changes can be induced by *H*. *pylori* infection. Some reports have shown the association between *H*. *pylori* infection and the degree of carotid atherosclerosis, but not with that of coronary atherosclerosis [39–41]. To our knowledge, this is the first study that employed the CLO test to detect current *H*. *pylori* infection, and linked this test with direct visualization of the coronary artery with cardiac MDCT in a relatively large number of healthy subjects. The significant correlation between *H*. *pylori* infection and subclinical coronary atherosclerosis in this study might be able to support *H*. *pylori* eradication as a potential cardiovascular prevention strategy.

How *H*. *pylori* infection can induce atherosclerosis has not been clearly established yet [10,29]. In this study, HDL-cholesterol was lower and triglyceride was higher in the CLO-positive group. Some data suggest that dysregulated lipid metabolism, including a low HDL-cholesterol level, may accelerate atherosclerosis in patients with *H*. *pylori* infection [10,42–45]. *Helicobacter pylori* carries lipopolysaccharide and can upregulate certain cytokines in the host, such as tumor necrosis factor-α (TNF-α) [9,10,35]. Inhibition of lipoprotein lipase by cytokines, such as TNF-α, may mobilize lipids from the tissues [46], leading to low serum HDL-cholesterol levels [47]. Besides perturbing the lipid metabolism, *H*. *pylori* infection may also directly induce atherosclerosis. Endothelial dysfunction induced by vacuolating cytotoxin A secreted from *H*. *pylori* [48,49], molecular mimicry by the autoimmune response [50], enhanced systemic inflammation [51], oxidative stress [52], and platelet aggregation [53] by *H*. *pylori* are all potential mechanisms of atherosclerosis that have been reported to be directly induced by *H*. *pylori* infection. The possibility of *H*. *pylori* induced-atherosclerosis independent of HDL-cholesterol levels is also supported by our data showing that *H*. *pylori* itself is associated with a higher incidence of significant coronary artery stenosis despite adjusting for HDL-cholesterol level. This significant association between *H*. *pylori* and significant coronary artery stenosis, irrespective of disturbed HDL-cholesterol levels, suggests that *H*. *pylori* infection may aggravate coronary atherosclerosis through both lipid metabolism-dependent and independent pathways.

CACS has also been used for suggesting the association between *H*. *pylori* seropositivity and coronary atherosclerosis. In a previous study, the degree of coronary atherosclerosis assessed by CACS was significantly associated with *H*. *pylori* seropositivity [32]. In our study, the presence of coronary artery calcium (CACS >0) was significantly higher in the CLO-positive group. On the other hand, there were no statistical differences in the presence of severe coronary artery calcium (CACS >100) and the mean value of log(CACS + 1) between the CLO-positive and CLO-negative subjects. Considering that CACS is more specific in subjects older than 60 years [54,55], the relationship between CACS and *H*. *pylori* infection needs to be further investigated in a larger study population.

The link between *H*. *pylori* infection and increased arterial PWV has been documented previously [56,57]. As chronic *H*. *pylori* infection is thought to induce systemic inflammation through cytokines such as interleukin-6 [44,51], not only coronary but also peripheral vascular changes can occur [58]. However, the mean PWV was not different according to the CLO positivity in our study. These inconsistent results may possibly be because of the differences in the PWV measurement sites (heart-carotid vs. brachial-ankle) and differences in the study population, as well as the diagnostic tool for *H*. *pylori* infection [56,57]. In addition, considering the previous reports that bacterial DNA of *H*. *pylori* was identified in coronary atherosclerotic plaque [10,59,60], *H*. *pylori* might be able to aggravate coronary atherosclerosis by direct invasion into the coronary vessels preferentially compared to other vessels regardless of systemic inflammation.

Our study has several limitations. First, this study lacks the measurement of cytotoxin-associated gene-A (CagA), which has been known to determine the pathogenicity of *H*. *pylori* in atherosclerosis [61–63]. It would be necessary to investigate this in future studies. Other limitations of this study are characteristics of the study population. As Gangnam Severance hospital is located in an affluent part of the capital city, the demographic and the socio-economic characteristics might be different from the general population. The individuals in our study population were more likely to be male, aged and upper white-collar workers prominently [64,65]. Lastly, we could not discriminate the subjects who had *H*. *pylori* eradication treatment previously because this was not included in the questionnaire of the health check-up. Nevertheless, our study is powerful and unique in demonstrating the association between subclinical atherosclerosis and *H*. *pylori* infection compared to other studies because our dataset is composed of extensive data encompassing MDCT, PWV, and CLO-test, the results of which are difficult to gather comprehensively.

In conclusion, current *H*. *pylori* infection is associated with subclinical but significant coronary artery stenosis in a healthy population. This result suggests the possibility that *H*. *pylori* eradication might be worthwhile in preventing coronary artery disease. Further studies are warranted to investigate whether CLO test positivity could be a new marker for the assessment of future coronary artery disease risk and the underlying mechanism of how *H*. *pylori* infection may lead to coronary atherosclerosis.

## References

[1] Epstein SE, Zhou YF, Zhu J. Infection and atherosclerosis: emerging mechanistic paradigms. Circulation. 1999;100: e20–28. 10421626 10.1161/01.cir.100.4.e20

[2] Ayada K, Yokota K, Kobayashi K, Shoenfeld Y, Matsuura E, Oguma K. Chronic infections and atherosclerosis. Ann N Y Acad Sci. 2007;1108: 594–602. 17894024 10.1196/annals.1422.062

[3] Coskun S, Kasirga E, Yilmaz O, Bayindir P, Akil I, Yuksel H, et al Is Helicobacter pylori related to endothelial dysfunction during childhood? Pediatr Int. 2008;50: 150–153. doi: 10.1111/j.1442-200X.2008.02542.x 18353048 10.1111/j.1442-200X.2008.02542.x

[4] Wang F, Meng W, Wang B, Qiao L. Helicobacter pylori-induced gastric inflammation and gastric cancer. Cancer Lett. 2014;345: 196–202. doi: 10.1016/j.canlet.2013.08.016 23981572 10.1016/j.canlet.2013.08.016

[5] Yamaoka Y, Graham DY. Helicobacter pylori virulence and cancer pathogenesis. Future Oncol. 2014;10: 1487–1500. doi: 10.2217/fon.14.29 25052757 10.2217/fon.14.29 PMC4197059

[6] Mentis A, Lehours P, Megraud F. Epidemiology and Diagnosis of Helicobacter pylori infection. Helicobacter. 2015;20 Suppl 1: 1–7. 10.1111/hel.12250 26372818

[7] Blankenberg S, Rupprecht HJ, Bickel C, Hafner G, Meyer J. [The role of inflammation and infection in acute coronary syndrome]. Herz. 2001;26 Suppl 1: 9–18. 11349630 10.1007/pl00014035

[8] Rezaee-Zavareh MS, Tohidi M, Sabouri A, Ramezani-Binabaj M, Sadeghi-Ghahrodi M, Einollahi B. Infectious and coronary artery disease. ARYA Atheroscler. 2016;12: 41–49. 27114736 PMC4834180

[9] Kountouras J, Polyzos SA, Katsinelos P, Zeglinas C, Artemaki F, Tzivras D, et al Cardio-cerebrovascular disease and Helicobacter pylori-related metabolic syndrome: We consider eradication therapy as a potential cardio-cerebrovascular prevention strategy. Int J Cardiol. 2017;229: 17–18. doi: 10.1016/j.ijcard.2016.11.265 27887801 10.1016/j.ijcard.2016.11.265

[10] Manolakis A, Kapsoritakis AN, Potamianos SP. A review of the postulated mechanisms concerning the association of Helicobacter pylori with ischemic heart disease. Helicobacter. 2007;12: 287–297. doi: 10.1111/j.1523-5378.2007.00511.x 17669100 10.1111/j.1523-5378.2007.00511.x

[11] Sun J, Rangan P, Bhat SS, Liu L. A Meta-Analysis of the Association between Helicobacter pylori Infection and Risk of Coronary Heart Disease from Published Prospective Studies. Helicobacter. 2016;21: 11–23. doi: 10.1111/hel.12234 25997465 10.1111/hel.12234

[12] Tabata N, Sueta D, Akasaka T, Arima Y, Sakamoto K, Yamamoto E, et al Helicobacter pylori Seropositivity in Patients with Interleukin-1 Polymorphisms Is Significantly Associated with ST-Segment Elevation Myocardial Infarction. PLoS One. 2016;11: e0166240 doi: 10.1371/journal.pone.0166240 27832202 10.1371/journal.pone.0166240 PMC5104372

[13] Al-Nozha MM, Khalil MZ, Al-Mofleh IA, Al-Ghamdi AS. Lack of association of coronary artery disease with H.pylori infection. Saudi Med J. 2003;24: 1370–1373. 14710286

[14] Kanbay M, Gur G, Yucel M, Yilmaz U, Muderrisoglu H. Helicobacter pylori seroprevalence in patients with coronary artery disease. Dig Dis Sci. 2005;50: 2071–2074. doi: 10.1007/s10620-005-3009-7 16240217 10.1007/s10620-005-3009-7

[15] Quinn MJ, Foley JB, Mulvihill NT, Lee J, Crean PA, Walsh MJ, et al Helicobacter pylori serology in patients with angiographically documented coronary artery disease. Am J Cardiol. 1999;83: 1664–1666, A1666 10392873 10.1016/s0002-9149(99)00175-7

[16] Tsai CJ, Huang TY. Relation of Helicobacter pylori infection and angiographically demonstrated coronary artery disease. Dig Dis Sci. 2000;45: 1227–1232. 10877241 10.1023/a:1005522624004

[17] Chmiela M, Kowalewicz-Kulbat M, Miszczak A, Wisniewska M, Rechcinski T, Kolodziej K, et al A link between Helicobacter pylori and/or Chlamydia spp. infections and atherosclerosis. FEMS Immunol Med Microbiol. 2003;36: 187–192. 12738390 10.1016/S0928-8244(03)00030-0

[18] Kowalski M, Konturek PC, Pieniazek P, Karczewska E, Kluczka A, Grove R, et al Prevalence of Helicobacter pylori infection in coronary artery disease and effect of its eradication on coronary lumen reduction after percutaneous coronary angioplasty. Dig Liver Dis. 2001;33: 222–229. 11407666 10.1016/s1590-8658(01)80711-8

[19] Jin SW, Her SH, Lee JM, Yoon HJ, Moon SJ, Kim PJ, et al The association between current Helicobacter pylori infection and coronary artery disease. Korean J Intern Med. 2007;22: 152–156. doi: 10.3904/kjim.2007.22.3.152 17939331 10.3904/kjim.2007.22.3.152 PMC2687700

[20] Schottker B, Adamu MA, Weck MN, Muller H, Brenner H. Helicobacter pylori infection, chronic atrophic gastritis and major cardiovascular events: a population-based cohort study. Atherosclerosis. 2012;220: 569–574. doi: 10.1016/j.atherosclerosis.2011.11.029 22189198 10.1016/j.atherosclerosis.2011.11.029

[21] Yu XJ, Yang X, Feng L, Wang LL, Dong QJ. Association between Helicobacter pylori infection and angiographically demonstrated coronary artery disease: A meta-analysis. Exp Ther Med. 2017;13: 787–793. doi: 10.3892/etm.2017.4028 28352367 10.3892/etm.2017.4028 PMC5348668

[22] Kuller LH, Arnold AM, Psaty BM, Robbins JA, O’Leary DH, Tracy RP, et al 10-year follow-up of subclinical cardiovascular disease and risk of coronary heart disease in the Cardiovascular Health Study. Arch Intern Med. 2006;166: 71–78. doi: 10.1001/archinte.166.1.71 16401813 10.1001/archinte.166.1.71

[23] Kuller L, Borhani N, Furberg C, Gardin J, Manolio T, O’Leary D, et al Prevalence of subclinical atherosclerosis and cardiovascular disease and association with risk factors in the Cardiovascular Health Study. Am J Epidemiol. 1994;139: 1164–1179. 8209875 10.1093/oxfordjournals.aje.a116963

[24] Silverman MG, Harkness JR, Blankstein R, Budoff MJ, Agatston AS, Carr JJ, et al Baseline subclinical atherosclerosis burden and distribution are associated with frequency and mode of future coronary revascularization: multi-ethnic study of atherosclerosis. JACC Cardiovasc Imaging. 2014;7: 476–486. doi: 10.1016/j.jcmg.2014.03.005 24831208 10.1016/j.jcmg.2014.03.005 PMC4024837

[25] Chey WD, Wong BC, Practice Parameters Committee of the American College of G. American College of Gastroenterology guideline on the management of Helicobacter pylori infection. Am J Gastroenterol. 2007;102: 1808–1825. doi: 10.1111/j.1572-0241.2007.01393.x 17608775 10.1111/j.1572-0241.2007.01393.x

[26] Atkinson NS, Braden B. Helicobacter Pylori Infection: Diagnostic Strategies in Primary Diagnosis and After Therapy. Dig Dis Sci. 2016;61: 19–24. doi: 10.1007/s10620-015-3877-4 26391269 10.1007/s10620-015-3877-4

[27] Duntas LH, Wartofsky L. Cardiovascular risk and subclinical hypothyroidism: focus on lipids and new emerging risk factors. What is the evidence? Thyroid. 2007;17: 1075–1084. doi: 10.1089/thy.2007.0116 17900236 10.1089/thy.2007.0116

[28] Chon SJ, Heo JY, Yun BH, Jung YS, Seo SK. Serum Thyroid Stimulating Hormone Levels Are Associated with the Presence of Coronary Atherosclerosis in Healthy Postmenopausal Women. J Menopausal Med. 2016;22: 146–153. doi: 10.6118/jmm.2016.22.3.146 28119894 10.6118/jmm.2016.22.3.146 PMC5256366

[29] Liu M, Li XC, Lu L, Cao Y, Sun RR, Chen S, et al Cardiovascular disease and its relationship with chronic kidney disease. Eur Rev Med Pharmacol Sci. 2014;18: 2918–2926. 25339487

[30] Mason JC, Libby P. Cardiovascular disease in patients with chronic inflammation: mechanisms underlying premature cardiovascular events in rheumatologic conditions. Eur Heart J. 2015;36: 482–489c. doi: 10.1093/eurheartj/ehu403 25433021 10.1093/eurheartj/ehu403 PMC4340364

[31] de Ferranti S, Rifai N. C-reactive protein and cardiovascular disease: a review of risk prediction and interventions. Clin Chim Acta. 2002;317: 1–15. 11814453 10.1016/s0009-8981(01)00797-5

[32] Park MJ, Choi SH, Kim D, Kang SJ, Chung SJ, Choi SY, et al Association between Helicobacter pylori Seropositivity and the Coronary Artery Calcium Score in a Screening Population. Gut Liver. 2011;5: 321–327. doi: 10.5009/gnl.2011.5.3.321 21927661 10.5009/gnl.2011.5.3.321 PMC3166673

[33] Ikeda A, Iso H, Sasazuki S, Inoue M, Tsugane S, Group JS. The combination of Helicobacter pylori- and cytotoxin-associated gene-A seropositivity in relation to the risk of myocardial infarction in middle-aged Japanese: The Japan Public Health Center-based study. Atherosclerosis. 2013;230: 67–72. doi: 10.1016/j.atherosclerosis.2013.06.013 23958254 10.1016/j.atherosclerosis.2013.06.013

[34] Rogha M, Nikvarz M, Pourmoghaddas Z, Shirneshan K, Dadkhah D, Pourmoghaddas M. Is helicobacter pylori infection a risk factor for coronary heart disease? ARYA Atheroscler. 2012;8: 5–8. 23056092 PMC3448393

[35] Adiloglu AK, Can R, Kinay O, Aridogan BC. Infection with Chlamydia pneumoniae but not Helicobacter pylori is related to elevated apolipoprotein B levels. Acta Cardiol. 2005;60: 599–604. doi: 10.2143/AC.60.6.2004930 16385920 10.2143/AC.60.6.2004930

[36] Chmiela M, Gajewski A, Rudnicka K. Helicobacter pylori vs coronary heart disease—searching for connections. World J Cardiol. 2015;7: 187–203. doi: 10.4330/wjc.v7.i4.187 25914788 10.4330/wjc.v7.i4.187 PMC4404374

[37] Mendall MA, Goggin PM, Molineaux N, Levy J, Toosy T, Strachan D, et al Relation of Helicobacter pylori infection and coronary heart disease. Br Heart J. 1994;71: 437–439. 8011406 10.1136/hrt.71.5.437 PMC483719

[38] Libby P, Ridker PM, Hansson GK. Progress and challenges in translating the biology of atherosclerosis. Nature. 2011;473: 317–325. doi: 10.1038/nature10146 21593864 10.1038/nature10146

[39] Mete R, Oran M, Alpsoy S, Gunes H, Tulubas F, Turan C, et al Carotid intima-media thickness and serum paraoxonase-1 activity in patients with Helicobacter pylori. Eur Rev Med Pharmacol Sci. 2013;17: 2884–2889. 24254556

[40] Mayr M, Kiechl S, Mendall MA, Willeit J, Wick G, Xu Q. Increased risk of atherosclerosis is confined to CagA-positive Helicobacter pylori strains: prospective results from the Bruneck study. Stroke. 2003;34: 610–615. doi: 10.1161/01.STR.0000058481.82639.EF 12624280 10.1161/01.STR.0000058481.82639.EF

[41] Bao-Ge Q, Hui W, Yi-Guo J, Ji-Liang S, Zhong-Dong W, Ya-Fei W, et al The Correlation and Risk Factors between Carotid Intima-Media Thickening and Alcoholic Liver Disease Coupled with Helicobacter pylori Infection. Sci Rep. 2017;7: 43059 doi: 10.1038/srep43059 28220866 10.1038/srep43059 PMC5318877

[42] Niemela S, Karttunen T, Korhonen T, Laara E, Karttunen R, Ikaheimo M, et al Could Helicobacter pylori infection increase the risk of coronary heart disease by modifying serum lipid concentrations? Heart. 1996;75: 573–575. 8697159 10.1136/hrt.75.6.573 PMC484379

[43] Laurila A, Bloigu A, Nayha S, Hassi J, Leinonen M, Saikku P. Association of Helicobacter pylori infection with elevated serum lipids. Atherosclerosis. 1999;142: 207–210. 9920523 10.1016/s0021-9150(98)00194-4

[44] Pohjanen VM, Koivurova OP, Niemela SE, Karttunen RA, Karttunen TJ. Role of Helicobacter pylori and interleukin 6–174 gene polymorphism in dyslipidemia: a case-control study. BMJ Open. 2016;6: e009987 doi: 10.1136/bmjopen-2015-009987 26781506 10.1136/bmjopen-2015-009987 PMC4735314

[45] Chen LW, Chien CY, Hsieh CW, Chang LC, Huang MH, Huang WY, et al The Associations Between Helicobacter pylori Infection, Serum Vitamin D, and Metabolic Syndrome: A Community-Based Study. Medicine (Baltimore). 2016;95: e3616. 27149497 10.1097/MD.0000000000003616 PMC4863814

[46] Makoveichuk E, Vorrsjo E, Olivecrona T, Olivecrona G. TNF-alpha decreases lipoprotein lipase activity in 3T3-L1 adipocytes by up-regulation of angiopoietin-like protein 4. Biochim Biophys Acta. 2017;1862: 533–540. doi: 10.1016/j.bbalip.2017.02.005 28215713 10.1016/j.bbalip.2017.02.005

[47] Sheu WHH, Lee WJ, Chang RL, Chen YT. Plasma tumor necrosis factor alpha levels and insulin sensitivity in hypertensive subjects. Clin Exp Hypertens. 2000;22: 595–606. 10972164 10.1081/ceh-100100094

[48] Tobin NP, Henehan GT, Murphy RP, Atherton JC, Guinan AF, Kerrigan SW, et al Helicobacter pylori-induced inhibition of vascular endothelial cell functions: a role for VacA-dependent nitric oxide reduction. Am J Physiol Heart Circ Physiol. 2008;295: H1403–1413. doi: 10.1152/ajpheart.00240.2008 18660451 10.1152/ajpheart.00240.2008

[49] Diomedi M, Stanzione P, Sallustio F, Leone G, Renna A, Misaggi G, et al Cytotoxin-associated Gene-A-positive Helicobacter pylori strains infection increases the risk of recurrent atherosclerotic stroke. Helicobacter. 2008;13: 525–531. doi: 10.1111/j.1523-5378.2008.00627.x 19166418 10.1111/j.1523-5378.2008.00627.x

[50] Okada T, Ayada K, Usui S, Yokota K, Cui J, Kawahara Y, et al Antibodies against heat shock protein 60 derived from Helicobacter pylori: diagnostic implications in cardiovascular disease. J Autoimmun. 2007;29: 106–115. doi: 10.1016/j.jaut.2007.05.004 17606364 10.1016/j.jaut.2007.05.004

[51] Nakagawa H, Tamura T, Mitsuda Y, Goto Y, Kamiya Y, Kondo T, et al Significant association between serum interleukin-6 and Helicobacter pylori antibody levels among H. pylori-positive Japanese adults. Mediators Inflamm. 2013;2013: 142358 doi: 10.1155/2013/142358 24453409 10.1155/2013/142358 PMC3881527

[52] Nazligul Y, Aslan M, Horoz M, Celik Y, Dulger AC, Celik H, et al The effect on serum myeloperoxidase activity and oxidative status of eradication treatment in patients Helicobacter pylori infected. Clin Biochem. 2011;44: 647–649. doi: 10.1016/j.clinbiochem.2011.03.001 21396358 10.1016/j.clinbiochem.2011.03.001

[53] Zuin M, Rigatelli G, Del Favero G, Picariello C, Meggiato T, Conte L, et al Coronary artery disease and Helicobacter pylori infection: Should we consider eradication therapy as cardiovascular prevention strategy? Int J Cardiol. 2016;223: 711–712. doi: 10.1016/j.ijcard.2016.08.320 27573593 10.1016/j.ijcard.2016.08.320

[54] Budoff MJ, Georgiou D, Brody A, Agatston AS, Kennedy J, Wolfkiel C, et al Ultrafast computed tomography as a diagnostic modality in the detection of coronary artery disease: a multicenter study. Circulation. 1996;93: 898–904. 8598080 10.1161/01.cir.93.5.898

[55] Haberl R, Becker A, Leber A, Knez A, Becker C, Lang C, et al Correlation of coronary calcification and angiographically documented stenoses in patients with suspected coronary artery disease: results of 1,764 patients. J Am Coll Cardiol. 2001;37: 451–457. 11216962 10.1016/s0735-1097(00)01119-0

[56] Ohnishi M, Fukui M, Ishikawa T, Ohnishi N, Ishigami N, Yoshioka K, et al Helicobacter pylori infection and arterial stiffness in patients with type 2 diabetes mellitus. Metabolism. 2008;57: 1760–1764. doi: 10.1016/j.metabol.2008.08.001 19013302 10.1016/j.metabol.2008.08.001

[57] Adachi K, Arima N, Takashima T, Miyaoka Y, Yuki M, Ono M, et al Pulse-wave velocity and cardiovascular risk factors in subjects with Helicobacter pylori infection. J Gastroenterol Hepatol. 2003;18: 771–777. 12795747 10.1046/j.1440-1746.2003.03059.x

[58] Budzynski J, Wisniewska J, Ciecierski M, Kedzia A. Association between Bacterial Infection and Peripheral Vascular Disease: A Review. Int J Angiol. 2016;25: 3–13. doi: 10.1055/s-0035-1547385 26900306 10.1055/s-0035-1547385 PMC4760071

[59] Kowalski M, Rees W, Konturek PC, Grove R, Scheffold T, Meixner H, et al Detection of Helicobacter pylori specific DNA in human atheromatous coronary arteries and its association to prior myocardial infarction and unstable angina. Dig Liver Dis. 2002;34: 398–402. 12132786 10.1016/s1590-8658(02)80036-6

[60] Farsak B, Yildirir A, Akyon Y, Pinar A, Oc M, Boke E, et al Detection of Chlamydia pneumoniae and Helicobacter pylori DNA in human atherosclerotic plaques by PCR. J Clin Microbiol. 2000;38: 4408–4411. 11101572 10.1128/jcm.38.12.4408-4411.2000 PMC87613

[61] Crabtree JE, Kersulyte D, Li SD, Lindley IJ, Berg DE. Modulation of Helicobacter pylori induced interleukin-8 synthesis in gastric epithelial cells mediated by cag PAI encoded VirD4 homologue. J Clin Pathol. 1999;52: 653–657. 10655985 10.1136/jcp.52.9.653 PMC501539

[62] Huang B, Chen Y, Xie Q, Lin G, Wu Y, Feng Y, et al CagA-positive Helicobacter pylori strains enhanced coronary atherosclerosis by increasing serum OxLDL and HsCRP in patients with coronary heart disease. Dig Dis Sci. 2011;56: 109–114. doi: 10.1007/s10620-010-1274-6 20503072 10.1007/s10620-010-1274-6

[63] Franceschi F, Niccoli G, Ferrante G, Gasbarrini A, Baldi A, Candelli M, et al CagA antigen of Helicobacter pylori and coronary instability: insight from a clinico-pathological study and a meta-analysis of 4241 cases. Atherosclerosis. 2009;202: 535–542. doi: 10.1016/j.atherosclerosis.2008.04.051 18599062 10.1016/j.atherosclerosis.2008.04.051

[64] Kim DW, Park JC, Rim TT, Jung UW, Kim CS, Donos N, et al Socioeconomic disparities of periodontitis in Koreans based on the KNHANES IV. Oral Dis. 2014;20: 551–559. doi: 10.1111/odi.12168 24033864 10.1111/odi.12168

[65] Kim SK, Kim H, Lee K, Kang HT, Oh SS, Ko SB. The Relationship between Injury and Socioeconomic Status in Reference to the Fourth Korean National Health and Nutrition Examination Survey. Ann Occup Environ Med. 2014;26: 1 doi: 10.1186/2052-4374-26-1 24472308 10.1186/2052-4374-26-1 PMC3916067

